# Ototoxicity: A review of South African studies

**DOI:** 10.4102/safp.v63i1.5187

**Published:** 2021-03-15

**Authors:** Selvarani Moodley, Claudine Storbeck, Nomthandazo Gama

**Affiliations:** 1 Centre for Deaf Studies, Faculty of Humanities, University of the Witwatersrand, Johannesburg, South Africa

**Keywords:** ototoxicity, pharmacology, audiology, paediatric hearing loss, ototoxicity guidelines, knowledge

## Abstract

**Background:**

Ototoxicity is damage to cells in the inner ear after administering a toxic drug, with a resultant hearing loss. Drugs used to treat illnesses such as cancer, tuberculosis, human immuno-deficiency virus (HIV) and infections are potentially ototoxic. South Africa has one of the highest rates of HIV and tuberculosis, and thus a potentially greater degree of the population is being affected by hearing loss from the medications used to treat these illnesses.

**Methods:**

To determine the current status of research in ototoxicity, a systematic literature review was carried out to determine the focus areas of South African studies for the period 1989–2019. From the database search engines used (Science Direct, Ebscohost and Proquest), a total of 33 relevant articles were identified, including the themes of pharmacology, audiology and knowledge.

**Results:**

Studies were conducted in the three most resourced provinces in South Africa. Findings indicate that there is a need for educating doctors regarding ototoxicity and a delineation of the role of the audiologist in monitoring and management of ototoxic hearing loss. There is a resultant need for audiology training on the pharmacology of ototoxic medication, otoprotective strategies and adherence to recommended guidelines. This has implications for university audiology training programmes and curriculum planning. The need for development of South Africa-specific audiology guidelines was highlighted.

**Conclusion:**

Whilst it is noted that there is a lack of resources for effective implementation of ototoxicity-monitoring protocols, it is also noted that there are measures and otoprotective strategies that can be put in place without additional resources.

## Introduction

A confirmed cause of acquired hearing loss is potential drug ototoxicity from medications used to treat illnesses such as cancer, tuberculosis (TB), human immuno-deficiency virus (HIV) and serious infections.^1,2^ If the individual is susceptible to hearing loss these ototoxic drugs can cause permanent sensorineural damage to the cells in the internal ear.^3^ These drugs include aminoglycosides, platinum compound treatments and loop diuretics.^4^ As a preventative measure for acquired hearing loss from ototoxicity, hearing tests allow for early detection of hearing loss. This allows for the initiation of timely treatment and prevention of further loss of hearing, where possible.^5^

With the extensive use of potential ototoxic medication because of the high rates of HIV and TB in South Africa, it would be expected that ototoxicity monitoring and management are a standardised part of best practice procedure, conducted in accordance with South Africa-specific guidelines. However, there seems to be limited knowledge in this field, with limited focus on this topic in audiology training programmes.

To determine the current status of research and knowledge in the area of ototoxicity in South Africa, a systematic literature review was carried out to determine the focus areas of studies and where further research needs to be performed.

## Research methodology

### Research design

The aims of this review are to determine the current status of research and knowledge in the area of ototoxicity in South Africa.

The objectives of this study are:

- to detail the number of peer-reviewed articles on ototoxicity in South Africa from January 1989 to March 2019, and to classify the articles according to type of publication (theoretical or empirical) to provide a description of the current knowledge base on ototoxicity;
- to identify the themes of the articles according to the chronological time frame to obtain an overview of the areas of focus and progression of studies across the 30-year period; and
- to outline the geographic coverage of research conducted.

### Data collection

An electronic academic database search was conducted by using a combination of search terms (doctor’s knowledge + ototoxicity + South Africa, ototoxicity + South Africa) related to ototoxicity. The search parameters included only peer-reviewed articles (thus excluding theses and dissertations) published between January 1989 and March 2019. A Boolean search by using the specified search terms was conducted on the academic database search engines used for this research (Science Direct, EBSCOhost and ProQuest). Only articles on studies conducted in South Africa were included.

As all articles were published in academic journals, assessing article quality was not an inclusion criterion. Grey and unpublished literature was excluded, as commenting on the quality and accuracy of these types of documents was not within the scope or objectives of this research.

A total of 288 articles were identified from the 3 academic database search engines. Of the 288 articles, 257 were found to be not relevant. Exclusion criteria included: (1) a focus on a topic other than ototoxicity and (2) not South African research. Exclusion of irrelevant articles left a total of 31 articles. Five duplicates were removed, resulting in a total of 26 articles identified as relevant to the search. An additional seven articles were identified through perusal of reference lists of identified articles. Thus, a total of 33 articles relating to ototoxicity in South Africa were included in this review (Table 1). These 33 articles comprised 16 theoretical and 17 empirical studies conducted in the period of the search, that is 1989–2019.

**TABLE 1:** Description of the search results identifying articles for the review.

Procedural steps	Number of articles	Description
1. Database search results	288	Three databases (EBSCOhost, Science Direct and ProQuest)
2. Database results examined for scope review	31	A total of 288 titles and keywords were examined for relevance to the topic. Where there was uncertainty about relevance abstracts were reviewed. A total of 257 articles were excluded based on relevance.
3. Database search results – duplicates omitted	26	Five duplicates were omitted.
4. Identification of additional reports relevant to review	7	Reference lists of articles were reviewed to identify additional articles.
5. Reports included in review	33	Articles forming part of the final review

### Data analysis

Articles were read and classified according to the following:

- Type of research publication (theoretical or empirical)
- An identified theme that was arranged according to year of publication, so as to determine the chronological progression of studies on ototoxicity in South Africa
- Geographical area of studies

### Ethical considerations

This article was a review of publications and did not include human or animal subjects. There was thus no need for ethical clearance.

## Results

**FIGURE 1:**
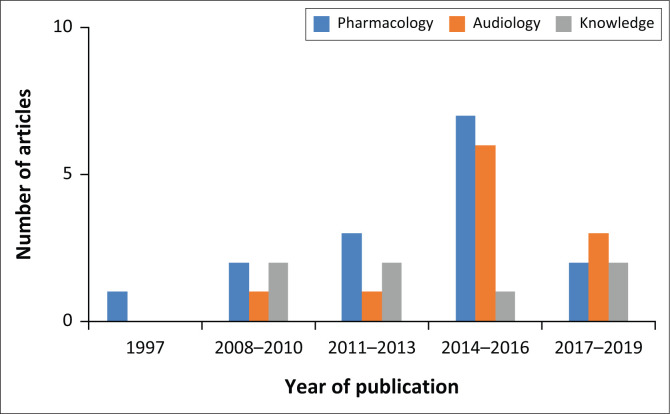
Chronological pattern of themes.

A total of 33 peer-reviewed articles relating to ototoxicity in South Africa from the period 1989 to 2019 were identified. A list and summary of articles that form part of this review is included in Appendix 1. Analysis of the article focus showed three themes in the published research: pharmacology, audiology and knowledge. Articles were grouped chronologically according to these identified themes (Figure 1).

The period of extensive research in ototoxicity began in 2008, with only one article published prior to this period (in 1997). This article was on the theme of pharmacology with a focus on genetics.^6^ Most of the studies conducted were in the areas of pharmacology (15, 46%) and audiology (11, 33%), with a few (7, 21%) focussing on knowledge of ototoxicity.

**FIGURE 2:**
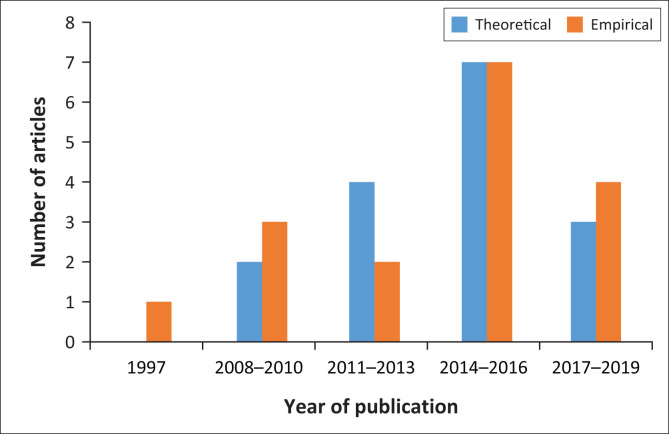
Chronological pattern of research type.

The number of theoretical articles shows a steady increase over the years. Empirical studies on ototoxicity are less consistent in number, with a decrease in the 2011–2013 period compared with the 2008–2010 period. Parallel to this is the large increase in theoretical articles in the 2014–2016 period. Figure 2 shows the breakdown of research type classification according to chronological period in which research was carried out.

**FIGURE 3:**
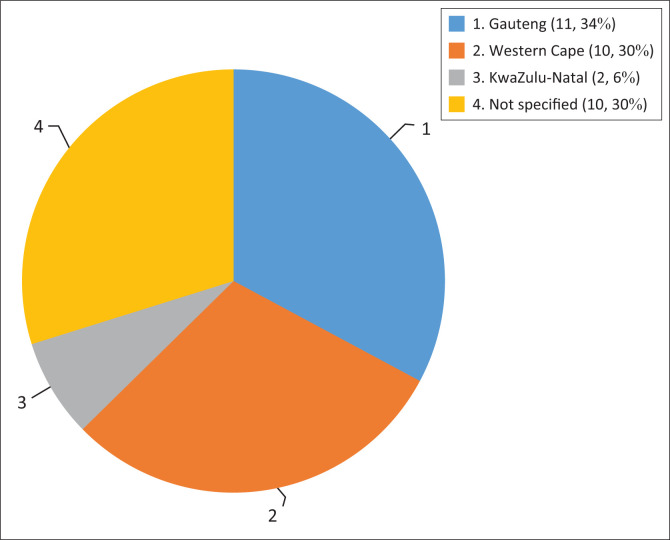
Geographical areas of studies.

A few studies identified provinces where research was conducted as shown in Figure 3. A significant number of studies (30%) did not specify region within South Africa in which the research was conducted.

## Discussion

The 33 articles included in this narrative review will be discussed chronologically and by theme. A large majority of the research were on the theme of pharmacology (46%), whereas the least number of studies focussed on knowledge (21%).

The pharmacology and biological bases of ototoxicity were identified as the initial focus of the studies reviewed.

### Pharmacology

Almost 50% of the articles were on the theme of pharmacology, with one article in 1997 and 14 articles published between 2009 and 2018. Nine of the 15 pharmacology articles (60%) focussed on aminoglycoside-induced ototoxicity.^6,7,8,9,10,11,12,13,14^ Aminoglycosides are broad-spectrum, bactericidal antibiotics that are used primarily for treating infections that are caused by Gram-negative pathogens.^15^

Two pharmacology-themed articles focussed on the link to genetics. Gardner et al.^6^ focussed on the genetic mutation causing sensorineural hearing loss in a family, following the use of streptomycin for the treatment of TB. The mitochondrial inheritance pattern and likeliness of maternal inheritance is highlighted, together with the importance of educating the family on ototoxicity. Bardien et al.^7^ also acknowledge the presence of specific mitochondrial DNA mutations, noting that these mutations typically remain inactive until exposed to aminoglycoside antibiotics. Genetic testing of patients at risk for aminoglycoside-induced hearing loss is recommended as a strategy for preservation of hearing, and thus a reduction of acquired social and economic consequences of acquired hearing loss from aminoglycoside ototoxicity. The use of genetic testing may be impractical in a developing country setting, where the majority of the population relies on limited resources in public healthcare settings. However, this could be applied in private healthcare settings, with research to determine the possible benefits and cost implications for implementation in public healthcare settings.

A review article on HIV and the need for ototoxicity monitoring^8^ precedes later articles focussing on HIV with multidrug-resistant tuberculosis (MDR-TB) and aminoglycosides. The primary effects of HIV and the increased risk of opportunistic infection affecting auditory function, as well as the need for large-scale studies on the effects of antiretroviral medication, are highlighted. Two later articles focus on HIV and use of aminoglycosides in the treatment of MDR-TB.^9,14^ Both articles (with the Hong et al.^14^ article including a meta-analysis of articles on aminoglycosides) concluded that MDR-TB patients who were HIV positive had a higher risk of aminoglycoside-induced hearing loss, with further studies needed to determine why there is increased ototoxicity in HIV-positive patients.

The 2015 article by Petersen and Rogers^11^ was a review of aminoglycoside-induced cochlear ototoxicity, with another article in the same year evaluating the safety and efficacy of using N-acetylcysteine in preventing aminoglycoside-induced ototoxicity.^12^ Petersen and Rogers^11^ mention otoprotectants, but they acknowledge that there is no commercially available medication to prevent cochleotoxicity. Kranzer et al.^12^ report that co-administration of N-acetylcysteine reduces the risk of ototoxicity by 80% and was not found to interact with MDR-TB treatment.

One article focussed on the use of aminoglycosides (amikacin) in neonates.^10^ Therapy of longer than 10 days, prior or current treatment with ototoxic drugs and presence of renal impairment is highlighted as increasing the risk of ototoxicity. Careful monitoring by using high-frequency otoacoustic emission testing is recommended.

The use of cisplatins and associated ototoxicity was the focus of 3 of the 14 (21%) pharmacology articles.^16,17,18^ Phanguphangu and Ramma^17^ found a high incidence of cisplatin-induced ototoxicity in paediatric cancer patients and highlight the importance of early identification and intervention. Adult patients with lymphoma and head and neck tumours with a cumulative dosage of cisplatin were found to have increased risk of hearing loss.^16^

Paken et al.’s^18^ article was a review of cisplatin-associated ototoxicity for the health professional. This review was preceded by a university implementing a service learning approach. Pharmacology lectures facilitated by the Pharmacy Department were included in health audiology lectures, so as to improve student audiologists’ knowledge of pharmacology as related to ototoxicity.

### Knowledge

The theme of ototoxicity knowledge mainly focusses on doctors’ knowledge, except for one article that included parents’ knowledge.^19^ The first study related to ototoxicity knowledge was a pilot study conducted in Gauteng (and focussed on awareness of oncologists). Although doctors had some form of awareness of the effects of ototoxic drugs, patients were not given specific information regarding ototoxic effects from prescribed medication.^20^ The article reveals a gap in knowledge on the different levels of ototoxicity and the role of audiologists, highlighting that there was no protocol on the frequency of audiological monitoring of patients undergoing chemotherapy.^20^

The article following on from this^21^ focused on healthcare workers’ knowledge of ototoxicity with a focus on TB. As with the 2009 article,^20^ this study revealed a lack of awareness of ototoxicity and a lack of collaboration with audiologists. The authors highlighted the need for education of patients and healthcare workers and the need for involvement of audiologists in the establishment of ototoxicity monitoring programmes.^21^ The focus on healthcare workers’ knowledge then expanded to include general practitioners’ knowledge, as well as a focus on ototoxicity monitoring.^22^ Although previous studies reviewed stressed the need for audiological monitoring as a recommendation, this study was the first to assess and confirm that audiological monitoring was still not occurring. The research focus in 2016 was on the awareness, roles and duties within the field of ototoxicity management. The article revealed that disclosure of ototoxicity risks was limited, and although doctors regarded the audiologist as an important team member, very few referred their patients for audiological ototoxicity monitoring.^4^

In 2017, the focus of ototoxicity knowledge expanded once again to include parents’ awareness of ototoxicity,^19^ whilst still including further studies on doctors’ knowledge.^23^ Moroe and Hughes^19^ found that 45% of parents were not informed by the oncologist of the effects of ototoxic medication. Children were prescribed a combination of ototoxic medications (cisplatin and cyclophosphamide), further increasing the risk for hearing loss. Co-administration of two ototoxic medications increases the pharmacological risk of an acquired hearing loss^24^ and the practice of this co-administration might be an indication of doctors’ limited awareness of the pharmacotherapy of ototoxic drugs. A concern highlighted was the lack of improvement in audiological involvement during children’s chemotherapy treatment and co-administration of potentially ototoxic medication.^19^

Smith et al.^23^ focussed on doctor’s knowledge of pharmacotherapy-induced ototoxicity. Doctors in the oncology ward of a Gauteng hospital completed a survey to assess their knowledge on ototoxicity. Results indicated that practitioners had a good overall knowledge of ototoxicity and medication, but had less knowledge of ototoxicity monitoring.^23^

Thus, as late as 2017 ensuring the involvement of audiologists for monitoring of hearing was still a challenge. Ototoxicity monitoring involves a specific set of audiology guidelines to be followed to ensure early detection of a hearing loss from ototoxic medication. The inclusion of audiology-themed studies to determine current practice on ototoxic monitoring and management is thus important.

### Audiology

There were a total of 12 articles on the theme of audiology. Studies mainly focussed on audiological testing of adults (7, 58%) as opposed to children (inclusive of neonates) (3, 25%), with one study (8%) being nonspecific and two studies (17%) including a combination of adults and children. The majority of studies focussed on patients with HIV and/or TB, with only one study on cancer patients. Studies in the area of audiology that focussed on HIV were conducted in 2010, 2011, 2014 and 2016. Khoza-Shangase, in 2011,^25^ stressed the need for audiologists to be involved in the Food and Drug Administration (FDA) process that includes drug formulation, consent and monitoring of medication and adding the importance of audiologists working with other health professionals to ensure audiology monitoring. In 2014, Khoza-Shangase^26^ reiterated the essential role of audiologists in the production of medicines. The audiological focus results from the study that indicated the need to add higher frequencies when testing HIV patients for early identification of ototoxic effects (as standard audiological assessments do not include higher frequencies). Similar results were found in a study conducted by Appana, Joseph & Paken in 2016.^26^ The study conducted audiological testing on patients with MDR-TB receiving aminoglycoside treatment over 6 months at a hospital in KwaZulu-Natal. The results revealed that high frequencies were affected first, indicating the need for inclusion of high-frequency audiometry for the early detection of ototoxicity.

An additional focus area relating to ototoxic medication and audiology was the management of TB and the role of audiologists. A study focussing on MDR-TB looked at roles played by audiologists in managing patients with MDR-TB.^28^ The study found that 78% of audiologists knew about ototoxicity monitoring and 68% knew of the American Speech Hearing Association (ASHA) and American Academy of Audiology (AAA guidelines) for ototoxicity monitoring. However, it was noted in the study that South African audiologists did not comply with international standards of ototoxicity monitoring. The study found that 54% of respondents indicated modifying international guidelines to not conducting speech audiometry, only testing certain frequencies and not conducting immittance audiometry. This made it difficult to monitor the progression of ototoxicity and hindered the referral to specialised services and gathering of epidemiological data.^28^ The recommendations were that South African based guidelines are developed, and that the Department of Health and the Health Professions Council of South Africa (HPCSA) develop context-relevant ototoxicity-monitoring protocols.^28^

A study looking at hearing loss among children treated for MDR-TB found that over 50% of the children who were treated for TB had a hearing loss.^5^ Concurring with results from the Seddon et al.^5^ study, a 2015 study on children with TB found that 48% of children taking potentially ototoxic medication had a hearing loss.^29^ However, it is acknowledged that a baseline assessment was not carried out, so the hearing status prior to receiving the ototoxic medication was not known. The study reiterated the need for ototoxicity monitoring, as well as recommending monitoring of the middle ear as irregularity was observed.^29^

In 2016, two studies focussed on adults with TB.^27,30^ Appana et al.^27^ conducted audiological testing for patients with MDR-TB receiving aminoglycoside treatment over 6 months at a hospital in KwaZulu-Natal. Results showed that hearing loss occurred gradually, and the number of patients experiencing hearing loss increased over time. Patients firstly identified tinnitus and vertigo as ototoxic effects. It was noted that because of the gradual progression of hearing loss it is essential that there is audiological monitoring and evaluation, as well as provision of rehabilitation services.^27^

Khoza-Shangase and Stirk^30^ conducted a study in Gauteng, which is the most equipped province in the country. Of the five state hospitals included in the research, only one had an ototoxicity-monitoring programme, and from that only one hospital 66% of the sample enrolled in ototoxicity monitoring (Khoza-Shangase and Stirk, 2016). Findings from the study indicated that a baseline audiogram was carried out a month after the start of treatment, rather than at the recommended time of initiation of treatment. In addition, monitoring was carried out once a month rather than the recommended once or twice a week.^30^ The study found that there was still a need for South African guidelines on ototoxic monitoring and more audiologists as the number of patients far exceeded the number of specialists.^30^ In 2016, the issue of ototoxic monitoring and development of guidelines was still something South Africa was grappling with. The need for ototoxic monitoring that meets the South African context is reiterated in the conclusion by Khoza-Shangase in 2017. There is also further discussion of the important role (and need for involvement) of audiologists in the manufacturing of drugs and the creation of guidelines.^31^

There was one study that outlined the research protocol to be used in researching cisplatin-associated ototoxicity in patients receiving chemotherapy.^32^ This study outlined the protocol used in research that aimed to determine the feasibility of an audiological-monitoring programme in KwaZulu-Natal.

The latest identified study from the review search was conducted by Ramma, Nhokwara and Rogers.^33^ It was found that 90% of outpatients did not attend scheduled sessions for ototoxicity monitoring. Patients who attended sessions were those found to develop a hearing loss after treatment and those who received a baseline audiometric assessment within a month of treatment initiation. It is recommended that a baseline assessment be carried out within the recommended 72 h of initiation of treatment.

Research over the years has indicated that the monitoring of ototoxicity is not clearly understood by patients or professionals and is still an area that needs further development and training. This development and training include the compilation of South Africa-specific ototoxicity-monitoring guidelines and the training of doctors and audiologists on adherence to these guidelines.

## Conclusion

South Africa has one of the highest rates of MDR-TB in the world, placing a strain on the healthcare system and highlighting the need for ototoxicity monitoring.^28^ However, it is clear that ototoxicity monitoring is not occurring because of a lack of collaboration between relevant healthcare providers. The audiologist, Ear, Nose and Throught (ENT) specialist and doctor should be in communication regarding drug dosage, the manner of the drug’s absorption and excretion, risk factors and the patient’s audiologic status.^3^ It is also important to raise the awareness of doctors regarding ethical practice, including the need for disclosure to patients of the risks when prescribing ototoxic medication.^4^ With the high prevalence of HIV infection and likely increase in incidence of hearing loss, information on possible hearing loss should be included as part of the process of obtaining informed consent for the initiation of aminoglycoside therapy.^9^

Ototoxicity monitoring aims to detect hearing loss before speech frequencies are affected and communication problems develop.^9^ It is important to assess hearing thresholds beyond the 250 Hz to 8000 Hz frequency range when monitoring ototoxicity, with clinics investing in audiometers that have a high-frequency test capability and additional headphones to test at higher frequencies.^34^ However, audiologists are not adhering to internationally recommended guidelines. Audiologists are practising in a manner allowing for maximisation of resources in proving high-quality healthcare, but they are not engaging best practice guidelines.^28^ Studies in this review indicate a clear need for South Africa-specific guidelines based on international best practice.

The obstacles in implementing ototoxicity monitoring in developing countries include limited finances and budgets within the healthcare system, limited staff and a lack of services at small healthcare facilities.^9^ South African studies have indicated an increased need for educating doctors regarding ototoxicity and the role of the audiologist in monitoring and managing ototoxic hearing loss. These are measures and otoprotective strategies that can be put in place without any additional need for resources. However, it is also noted that audiologists need additional training on the pharmacology of ototoxic medication, otoprotective strategies and the adherence to recommended guidelines (including use of high-frequency audiometry). This will ensure that audiologists are an effective resource in the prevention, monitoring and management of ototoxic hearing loss. This acknowledgement of the need for training has important implications for university audiology training programme curriculum planning, as well as for continuing professional development activities.

All studies in this review were in the more resourced provinces of South Africa (Gauteng, KwaZulu-Natal and Western Cape). Even in the well-resourced provinces, patients were not enrolled in an ototoxicity-monitoring programme.^35^ Studies in other provinces (beyond the provinces identified in this review) are needed to obtain a clearer understanding of the knowledge and processes followed in the area of ototoxicity monitoring and management.

## Appendix 1

**TABLE 1-A1:** Articles included in review.

Title	Authors	Year of publication	Article type	Summary	Theme
Familial streptomycin ototoxicity in a South African family: A mitochondrial disorder	Gardner JC, Goliath R, Viljoen D, Sellars S, Cortopassi G, Hutchin T and Beighton P.	1997	Empirical	Investigates a family with streptomycin-induced hearing loss and links to a mitochondrial DNA mutation. The predisposition of the mutation is identified.	Pharmacology
Aminoglycoside-induced hearing loss: South Africans at risk	Bardien S, Jong GD, Schaaf HS, Harris T, Fagan J and Petersen L.	2009	Theoretical	Summarises risk factors associated with TB treatment including the effects on the cochlear and vestibular systems. The need for audiological monitoring is highlighted. The importance of assessing frequency of genetic mutations predisposing patients to ototoxicity from aminoglycosides in South Africa is emphasised.	Pharmacology
Ototoxic effects of tuberculosis treatments: How aware are patients?	Khoza-Shangase K, Anniah M and Precious MN.	2009	Empirical	Adults undergoing treatment for TB were interviewed on their awareness of ototoxicity. None of the patients were enrolled in an ototoxicity-monitoring programme. The study highlights the need for education of patients and the implementation of ototoxicity-monitoring programmes.	Knowledge
Perceptions of oncologists at 2 state hospitals in Gauteng regarding the ototoxic effects of cancer chemotherapy: A pilot study	De Andrade V, Khoza-Shangase K and Hajat F.	2009	Empirical	Investigates the knowledge of oncologists on ototoxicity and monitoring. The article reveals a gap in knowledge on the different levels of ototoxicity and the role of audiologists in ototoxicity monitoring and management.	Knowledge
Is there a need for ototoxicity monitoring in patients with HIV/AIDS?	Khoza-Shangase K.	2010	Theoretical	Discusses finding from reviewing literature on ototoxicity and HIV. The expected increase in ototoxicity amongst South Africans because of the increase of HIV cases is discussed, as is the levels of hearing loss caused by the different degrees of HIV infection.	Pharmacology
Highly active antiretroviral therapy: Does it sound toxic?	Khoza-Shangase K.	2011	Empirical	The auditory status of patients receiving highly active antiretroviral therapy was monitored in an outpatient clinic in Gauteng. Subclinical hearing function changes were found in the experimental group using otoacoustic emissions, with no significant changes in the control group using pure tone audiometry. The need for more sensitive audiological assessment tools during drug trials is highlighted.	Audiology
Aminoglycoside-induced hearing loss in HIV-positive and HIV-negative multidrug-resistant tuberculosis patients	Harris T, Bardien S, Schaaf HS, Petersen L, De Jong G and Fagan JJ.	2012	Empirical	Audiological testing was conducted on patients with MDR-TB in Cape Town. Human-immunodeficiency positive patients treated with aminoglycosides are more likely to develop hearing loss. Auditory testing and rehabilitation should be part of the standard treatment for patients with MDR-TB.	Pharmacology
An overview of pharmacotherapy-induced ototoxicity	Natalie S and Alida N.	2013	Theoretical	An overview of ototoxic medication, including pharmacology- and audiology-monitoring strategies.	Pharmacology
Hearing loss in children treated for multidrug-resistant tuberculosis	Seddon JA, Thee S, Jacobs K, Ebrahim A, Hesseling AC and Schaaf HS.	2013	Empirical	Discusses hearing loss caused by MDR-TB medications, with a brief introduction on the effect of aminoglycosides. Children between 0 and 15 years being treated for MDR-TB had audiological testing. Over half of the study participants had a hearing loss.	Audiology
Ototoxicity in tuberculosis treatment in South Africa: Exploring the current status	Khoza-Shangase K.	2013	Empirical	Healthcare workers’ knowledge and management of TB-induced ototoxicity was researched, with results indicating a lack of awareness of ototoxicity. The need for education of patients and healthcare workers is highlighted. Audiologists should be more involved in the establishment of ototoxicity-monitoring programmes.	Knowledge
Ototoxicity monitoring in general medical practice: Exploring perceptions and practices of general practitioners about drug-induced auditory symptoms	Khoza-Shangase K and Jina K.	2013	Empirical	Explored doctors’ knowledge on ototoxicity monitoring and management in Gauteng. Although doctors have access to audiological services, not all utilise the service. Whilst aware of their role in ototoxicity monitoring, there is a lack of monitoring strategies for various reasons, including a symptomatic management protocol.	Knowledge
Use of amikacin in neonates and related ototoxicity	Engler D, Schellack N, Naude A and Gous AGS.	2013	Theoretical	Focusses on the effects of Amikacin administered to neonates. It discusses the movement from the time a drug is administered to excretion, including the side effects. Audiological screening with the use of otoacoustic emissions (OAEs) is discussed.	Pharmacology
High prevalence of cisplatin-induced ototoxicity in Cape Town, South Africa	Whitehorn H, Sibanda M, Lacerda M, Spracklen T, Ramma L, Dalvie S and Ramesar R.	2014	Theoretical	High doses of cisplatin, the first-line treatment of soft tissue cancer, resulted in increased risk of ototoxicity. South African patients may be at higher risk than patients of European or South American origin. Oncologists have become more aware of ototoxicity and the need for monitoring since 2006, but are still not routinely referring patients for audiological monitoring.	Pharmacology
Pharmaco-audiology vigilance and highly active antiretroviral therapy (HAART) in South Africa: Ototoxicity monitoring pursued	Khoza-Shangase K.	2014	Empirical	The auditory status of HIV-positive patients receiving two different regimens of HAART was monitored at a hospital in Johannesburg. The ototoxic nature of HAART is indicated and highlights the need for ototoxicity monitoring.	Pharmacology
A systematic review and meta-analysis of the efficacy and safety of N-acetylcysteine in preventing aminoglycoside-induced ototoxicity: Implications for the treatment of multidrug-resistant TB	Kranzer K, Elamin WF, Cox H, Seddon JA, Ford N and Drobniewski F.	2015	Theoretical	Reviews the use of N-acetylcysteine with aminoglycosides in preventing ototoxicity. Co-administration of N-acetylcysteine reduces the ototoxicity of aminoglycosides by 80% and may be effective in reducing ototoxicity in patients with MDR-TB.	Pharmacology
Aminoglycoside-induced hearing deficits: A review of cochlear ototoxicity	Petersen L and Rogers C.	2015	Theoretical	Reviews on the effect of aminoglycosides on hearing, as well as describes the monitoring and management of cochlear ototoxicity.	Pharmacology
Establishing a pharmacotherapy-induced ototoxicity programme within a service-learning approach	Schellack N, Wium AM, Ehlert K, Van Aswegen Y and Gous A.	2015	Theoretical	The implementation of an ototoxicity-monitoring curriculum at Sefako Makgatho University as developed and coordinated by the pharmacy department.	Pharmacology
Practices employed by audiologists in the management of adult patients with multidrug-resistant tuberculosis in South Africa	Govender M and Paken J.	2015	Empirical	Audiologists were surveyed on their audiological practice in the assessment and management of patients with MDR-TB in South Africa. There are no ototoxicity-monitoring guidelines in South Africa, and so audiologists modify international guidelines. The development of context-relevant monitoring guidelines is necessary.	Audiology
The occurrence of auditory dysfunction in children with TB receiving ototoxic medication at a TB hospital in South Africa	Ghafari N, Rogers C, Petersen L and Singh SA.	2015	Empirical	The auditory profiles of children receiving ototoxic medication at a TB hospital in the Cape Town metropolitan area are described. The average of higher thresholds (4000, 600 and 8000 Hz) should be used for classification of hearing loss in children receiving ototoxic medication.	Audiology
An audiological profile of patients infected with multi-drug resistant tuberculosis at a district hospital in KwaZulu-Natal	Appana D, Joseph L and Paken J.	2016	Empirical	Descriptive study of the incidence and nature of hearing loss amongst MDR-TB patients receiving aminoglycosides at a hospital in KwaZulu-Natal. High frequencies were affected first, indicating the need for inclusion of high-frequency audiometry for the early detection of ototoxicity.	Audiology
Audiological testing for ototoxicity monitoring in adults with tuberculosis in state hospitals in Gauteng, South Africa	Khoza-Shangase K and Stirk M.	2016	Theoretical	Focusses on ototoxicity amongst adult patients with TB. Audiological testing data are presented to understand the levels and procedures of ototoxicity monitoring in Gauteng hospitals.	Audiology
Cisplatin-associated ototoxicity: A review for the health professional	Paken J, Govender CD, Pillay M and Sewram V.	2016	Theoretical	A review of the ototoxic process from the use of cisplatin, including risk factors, is discussed. Ototoxic monitoring and stages at which it should occur is presented.	Pharmacology
Ototoxicity management: An investigation into doctors’ knowledge and practices, and the roles of audiologists in a tertiary hospital	Wium A and Gerber B.	2016	Empirical	Doctors’ knowledge on ototoxicity and ototoxicity disclosure to patients was determined via a survey at a tertiary hospital in a township context. Disclosure of ototoxicity risks was limited, and very few doctors referred their patients for audiologic ototoxicity monitoring.	Knowledge
Screening and monitoring of pharmacotherapy-induced ototoxicity in patients at Dr George Mukhari Academic Hospital	Van Aswegen Y, Schellack N, Gous AGS, Ehlert K and Phil AMWD.	2016	Empirical	Auditory testing of patients on ototoxic medication revealed a high-frequency hearing loss in the majority of patients treated with aminoglycosides and platinum compounds and loop diuretics. The study highlights the need for a multidisciplinary collaboration between the pharmacist and audiologist and the implementation of ototoxicity-monitoring programmes.	Audiology
Screening for possible nevirapine-induced ototoxicity in neonates, when administered as part of the prevention of mother to child transmission at Dr George Mukhari Academic hospital	Fourie J Schellack N, Engler D, Ehlert K, Wium AM. and Moagi T.	2016	Empirical	Nevirapine is used as a drug for the prevention of mother-to-child transmission of HIV. Administration of nevirapine was not associated with hearing loss, although further monitoring was suggested.	Audiology
The safety and tolerability of the second-line injectable antituberculosis drugs in children	Garcia-Prats AJ, Schaaf HS and Hesseling AC.	2016	Theoretical	Second-line injectable medications such as amikacin, kanamycin and capreomycin are ototoxic in at least 20% of children, as well as possibly causing electrolyte abnormalities, renal dysfunction and painful injection sites. Potential strategies for reducing adverse effects are presented.	Pharmacology
Ototoxic hearing loss in paediatrics-knowledge and perceptions amongst medical professionals	Smith N, Schellack N, Ehlert K and Gous AG.	2017	Empirical	Doctors in the oncology ward of a Gauteng hospital completed a survey to assess their knowledge on ototoxicity. Practitioners had a good overall knowledge of ototoxicity and medication, but less knowledge on ototoxicity monitoring.	Knowledge
Parents are aware of the ototoxic effects of chemotherapy in paediatrics undergoing cancer treatment – Professional versus parental views: A pilot study	Moroe NF and Hughes K.	2017	Empirical	Explores parents’ knowledge of the ototoxic effects of chemotherapy on their children and whether doctors inform parents of these effects.	Knowledge
Research protocol: Cisplatin associated ototoxicity amongst patients receiving cancer chemotherapy and feasibility of an audiological monitoring program	Paken J, Govender CD and Sewram V.	2017	Empirical	Describes the research protocol to be used for assessment of knowledge of healthcare workers on ototoxicity, as well as audiological assessment of patients treated with cisplatin for cervical cancer in KwaZulu-Natal.	Audiology
Risk versus benefit: Who assesses this in the management of patients on ototoxic drugs?	Khoza-Shangase K.	2017	Theoretical	Highlights the risk of ototoxicity and process involved in the monitoring of adverse drugs. The role of audiologists as representing patients during drug research and administration and providing information on ototoxic medication to patients is discussed.	Audiology
High incidence of cisplatin-induced ototoxicity in paediatric patients in the Western Cape, South Africa	Phanguphangu M and Ramma L.	2018	Empirical	Aimed to determine the incidence of hearing loss amongst paediatric cancer patients receiving cisplatin treatment. As much as 80% of patients developed a hearing loss, highlighting the need for early identification and appropriate intervention.	Pharmacology
Increased risk of aminoglycoside-induced hearing loss in MDR-TB patients with HIV coinfection	Hong H, Budhathoki C and Farley JE.	2018	Theoretical	Evaluation of the impact of simultaneous infection amongst patients with HIV and MDR-TB on aminoglycoside-induced hearing loss. Individuals with both MDR-TB and HIV-co-infection had a high risk of aminoglycoside-induced hearing loss, compared with non-HIV-infected individuals.	Pharmacology
Statistical factors associated with utilisation of ototoxicity monitoring services for multi-drug-resistant tuberculosis patients in the Western Cape	Ramma L, Nhokwara PT, and Rogers C.	2019	Theoretical	Discusses the use of aminoglycoside antibiotic and its effects amongst patients with MDR-TB, as well as ototoxicity monitoring in South Africa. The study further identifies what determinants affect the use of ototoxicity monitoring amongst outpatients receiving MDR-TB treatment in South Africa.	Audiology

DNA, deoxyribonucleic acid; TB, tuberculosis; MDR-TB, multidrug resistant tuberculosis; HAART, highly active antiretroviral therapy; HIV, human immuno-deficiency virus.
